# Towards an Ultra-Sensitive Temperature Sensor for Uncooled Infrared Sensing in CMOS–MEMS Technology

**DOI:** 10.3390/mi10020108

**Published:** 2019-02-06

**Authors:** Hasan Göktaş

**Affiliations:** Electrical and Electronic Engineering, Harran University, Şanlıurfa 63000, Turkey; hgoktas.gwu@gmail.com; Tel.: +90-414-318-3000

**Keywords:** CMOS, MEMS, microresonators, microelectromechanical systems, thermal detector, temperature sensor, infrared sensor, microbolometer

## Abstract

Microbolometers and photon detectors are two main technologies to address the needs in Infrared Sensing applications. While the microbolometers in both complementary metal-oxide semiconductor (CMOS) and Micro-Electro-Mechanical Systems (MEMS) technology offer many advantages over photon detectors, they still suffer from nonlinearity and relatively low temperature sensitivity. This paper not only offers a reliable solution to solve the nonlinearity problem but also demonstrate a noticeable potential to build ultra-sensitive CMOS–MEMS temperature sensor for infrared (IR) sensing applications. The possibility of a 31× improvement in the total absolute frequency shift with respect to ambient temperature change is verified via both COMSOL (multiphysics solver) and theory. Nonlinearity problem is resolved by an operating temperature sensor around the beam bending point. The effect of both pull-in force and dimensional change is analyzed in depth, and a drastic increase in performance is achieved when the applied pull-in force between adjacent beams is kept as small as possible. The optimum structure is derived with a length of 57 µm and a thickness of 1 µm while avoiding critical temperature and, consequently, device failure. Moreover, a good match between theory and COMSOL is demonstrated, and this can be used as a guidance to build state-of-the-art designs.

## 1. Introduction

Microbolometers offer many advantages with their compact size, low power, capability of working at room temperature, small cost, reliable and simpler fabrication technique over bulky or relatively expensive detectors (liquid-nitrogen cooled HgCdTe (MCT), [1] etc.) in Infrared (IR) Sensing application. Ideal microbolometers should consist of high sensitivity temperature sensors and an IR absorbing layer. The IR absorbing layer converts the incident radiation into heat, and that heat is converted into the electrical signal via a temperature sensor (non-resonant [2,3], resonant-sensing [4,5,6,7,8,9]). The resonant-sensing type sensor has many advantages over the non-resonant type, such as smaller dimension and relatively low noise, due to a high-quality factor of 2.4 × 10^6^ [9] and 1 million [10]. That is why resonant-sensing type sensors are also popular in mass sensing [11,12,13], but are mostly fabricated in Micro-Electro-Mechanical Systems (MEMS) technology (MEMS resonators) [5,6,7,8,9] rather than in complementary metal-oxide semiconductor (CMOS) technology (CMOS-MEMS resonators [4,14]).

A high-density focal plane array (FPAs) are very demanding for high-quality thermal imaging, and this requires a high-density integrated circuit (IC). It can be achieved by either building thermal detectors and IC on the same chip (CMOS–MEMS) [15,16] or bonding a separate IC and MEMS chip together [17]; however, the one that requires bonding brings extra fabrication costs and complexity. That is why CMOS–MEMS resonant-sensing type uncooled IR detectors are becoming more attractive, as they offer all-in-one (IC + MEMS), cost-effective and high sensitivity solution together. The main performance parameter for resonant-sensing type temperature sensors (cantilever, tuning fork, free–free beam, and fixed–fixed beam) is the temperature coefficient of frequency (TCF) that represents the magnitude of frequency shift (FS) with respect to the temperature change. The wide range frequency tuning capability of a fixed–fixed beam in comparison to other resonant-sensing types was demonstrated for the first time in [14] and later used in [4] to build a high sensitivity temperature sensor in CMOS technology. Moreover, fixed–fixed beam type CMOS–MEMS resonator [4] has the potential to offer high performance with their relatively high TCF (4537 ppm/K (Table 1)), while enabling a more reliable and simpler fabrication process. Despite their relatively large TCF, fixed–fixed beam type CMOS–MEMS resonators suffer from a nonlinearity problem and still need to have larger TCF for ultra-sensitive uncooled IR detection application.

In this work, the nonlinearity problem of fixed–fixed type CMOS–MEMS resonator is resolved by operating the resonator around the beam bending point. In addition, at least 31× (343 kHz/11 kHz) improvement in total absolute FS with an absolute |TCF| > 589,698 ppm/K are achieved according to COMSOL and theory for 57 µm long CMOS–MEMS resonator. |TCF| increases from 589,698 ppm/K to 2178,946 ppm/K when applied Joule-heating (Vth) changes from 3.3252 V to 3.3476 V according to COMSOL. Here both Joule-heating and the change in the ambient temperature are applied together in contrast to [4], where only the ambient temperature change was used to derive |TCF|. Moreover, the effect of the pull-in force between two adjacent beams is studied in detail to find the optimum resonator working parameters for the sake of larger |TCF|. The |TCF| drastically decreases from 2,333,771 ppm/K to 16,185 ppm/K when pull-in force increases from 7 MPa to 10,000 MPa according to COMSOL for 120 µm long CMOS–MEMS resonator due to decreases in thermal stress on both fixed ends. In addition, in contrast to [4], there is no thickness effect on FS while a shorter beam results in larger FS where the beam just starts to bend. The maximum temperature around beam bending point for 57 μm long beam is calculated as 530 K via COMSOL, and that does not exceed the maximum allowable temperature in CMOS–MEMS technology [18]. According to COMSOL and theory, a significant improvement in |TCF| for 57 µm long CMOS–MEMS resonator over previous works can be achieved (Table 1)

## 2. Fabrication

The CMOS–MEMS resonators can be fabricated via a post-process followed after a CMOS 0.6 µm process that includes a CHF_3_/O_2_ process for SiO_2_ etching between adjacent beams and XeF_2_ process for Silicon etching underneath the beams [14]. 

**Figure 1:**
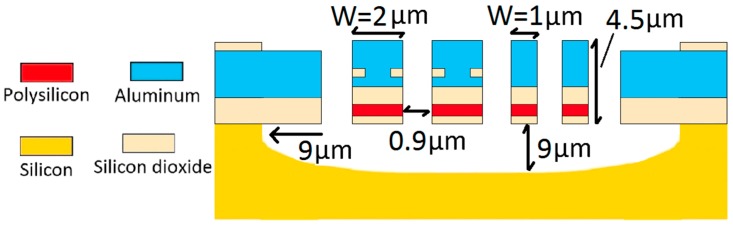
The cross section for Device 1 (W = 2 µm) and for Device 2 (W = 1 µm), where W is the thickness.

In this study, the device structures (Figure 1) are slightly changed for the sake of better performance. However, the distance between devices and the silicon etching ratio is kept the same.

## 3. Theory Modeling and Optimization

The working principle of the CMOS–MEMS resonator (Figure 1) is based on pull-in force (via DC bending voltage (Vdc) applied between two adjacent beams), and the Joule-heating voltage (Vth) applied on the embedded heater (polysilicon layer) through the resonant beam. Pull-in force enables the softening effect on the resonant beam and, consequently, starts the resonance operation while Joule-heating increases the temperature throughout the resonant beam and resultes in relatively high thermal stress on the fixed ends. This Joule-heating effect causes a wide range of frequency tuning and this was first time demonstrated in [14]. The resonance frequency with respect to axial load [19] is:

(1) $$f=\;\frac{4.73^{2}}{2\pi L^{2}}\;{\left(1+\frac{PL^{2}}{EI\pi^{2}}\right)}^{\frac{1}{2}}{\left(\frac{EI}{m}\right)}^{\frac{1}{2}}\left(1\right)$$

where *I* is the moment of inertia, *L* (m) is the length, *m* (kg/m) is the mass per unit length, and *P* is the total compressive axial load on fixed ends [20]. More detail is given in [18]. In addition to Equation (1), COMSOL was used to build the CMOS–MEMS resonators (Figure 1) and calculate their resonance frequency responses with respect to temperature. The simulation environment was selected as a vacuum, and ambient temperature (Tamb) was set to 273 K. Solid mechanics, heat transfer, and electric currents tools were combined together in multiphysics to couple heat transfer with solid mechanics and electric currents. Mesh study was conducted to find the optimum mesh set up for the simulation. Both the “extremely fine mesh” and “fine mesh” were compared to decrease time budget, where tetrahedral meshing was used throughout the structure. There was only a slight change observed between the results. Polysilicon conductance was set as 1.16 × 10^5^ S/m as it was already measured and verified [18]. Electric current was used to heat the beams via Joule-heating while the heat transfer module was used to model temperature distribution throughout the beam and solid mechanics was used to model deformation and mode shapes.

**Figure 2:**
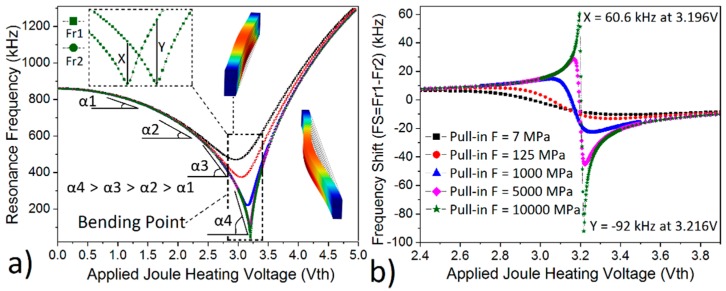
The effect of pull-in force (F) on the (**a**) Frequency tuning and (**b**) frequency shift (FS) in COMSOL simulation for Device 1 for a length of 120 µm long fixed–fixed beam, where Fr1 and Fr2 are the resonance frequency responses with ambient temperature of Tamb and Tamb + 1 K respectively.

The resonance frequency tuning range with the application of Joule-heating was around 761 kHz when the pull-in force was 7 MPa, and it was around 276.5 kHz when it was 10,000 MPa (Figure 2a). This is attributed to the fact that both the pull-in force and Joule-heating results in beam bending. Pull-in force, however, created an ignorable stress on the fixed ends in comparison to Joule-heating and consequently results in a very small frequency tuning range [21]. In another words, the bending should be resulted mainly because of thermal stresses (Joule-heating) while keeping the pull-in force as minimum as possible to get the maximum frequency tuning range.

The slope of the resonance frequency with respect to the applied Joule-heating voltage (Vth) was not constant but kept on increasing (α4 > α3 > α2 > α1) (Figure 2a) with an increase in temperature. This nonlinear effect was first observed in [14], and allows better FS at higher temperatures (Figure 2b) and consequently enables higher sensitivity temperature sensor design. This effect was analyzed partially in [4], and the temperature sensitivity was found as 2.98 kHz/C without any Joule-heating application.

**Figure 3:**
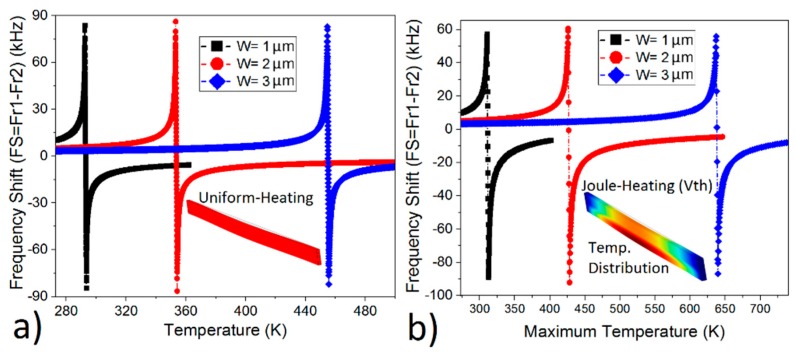
Frequency Shift (FS) with respect to 1 Kelvin (K) change by (**a**) Equation (1), and (**b**) COMSOL, when thickness (W) changes from 1 µm to 3 µm for Device 1 with a device length of 120 µm.

**Figure 4:**
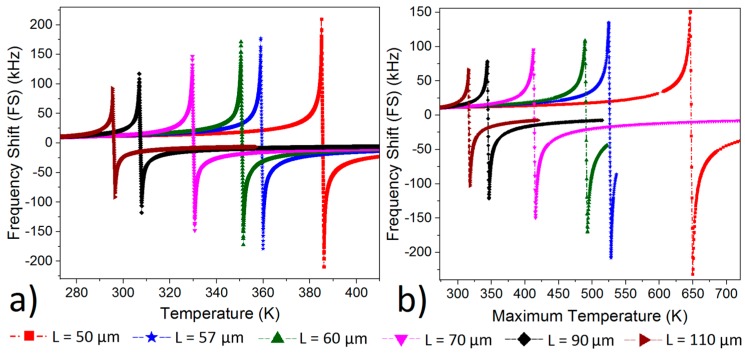
Frequency Shift (FS) with respect to 1 Kelvin (K) change by (**a**) Equation (1), and (**b**) COMSOL, when length (*L*) changes from 50 µm to 110 µm for Device 2 with a device thickness of 1 µm.

In contrast to [4,14], here we studied the FS in detail by combining both ambient temperature (Tamb) change and Joule-heating for highly sensitivity temperature sensors in microbolometer application. This required the full analysis of the frequency response (Figure 2a) where the resonance frequency decreases until it reaches the bending point and then starts to increase. Two different resonance frequency (Fr1, Fr2) responses with respect to applied Joule-heating voltage were calculated via COMSOL at two different environment temperature (Tamb1 = 273 K and Tamb2 = 274 K) in Figure 2, Figure 3 and Figure 4. Hence, FS for 1 Kelvin change can be derived by subtracting resonance frequency responses (Fs = Fr1−Fr2) for every applied Vth (Figure 2b, Figure 3 and Figure 4). The optimum device operation point (larger FS, consequently better sensitivity) was found around the bending point (Figure 2a) where the beam was just starting to have a 0.38 µm bending and gives maximum FS values (X and Y). The FS was 5 kHz when Vth = 0 V and reaches up to 60.6 kHz when Vth = 3.196 V and −92 kHz when Vth was 3.216 V. If Vth is switched from 3.196 V and 3.216 V, then total absolute FS will be X+|Y| = 152.6 kHz (Figure 2b). Moreover, the pull-in force should be as small as possible to create as sharp a bending curve as possible (Figure 2a). This, in turn, would result in a larger X and |Y| value and consequently larger FS and much better sensitivity. The total absolute FS was around 152.6 kHz (|TCF| = 2,333,771 ppm/K at Vth = 3.216 V) when pull-in force was 7 MPa whereas it was around 16.8 kHz (|TCF| = 16,185 ppm/K at Vth = 3.425 V) when pull-in force was 10,000 MPa. 

Further optimization was conducted by analyzing the dimensional effect to find the optimum structure for the sake of larger FS. The Joule-heating is studied in Figure 3b for Device 1 to study the effect of thickness on FS and in Figure 4b for Device 2 to study the effect of length on FS by using COMSOL. In the same way, uniform heating was applied to the beams to calculate the FS via Equation (1) in Figure 3a and Figure 4a. That is why max temperature is used to plot FS in Figure 3b and Figure 4b in contrast to the uniform temperature profile in Figure 3a and Figure 4a. The minimum pull-in force was applied to every beam in Figure 3 and Figure 4 to get the largest FS. A good match between COMSOL and Equation (1) is achieved for the total absolute FS. 

The CMOS–MEMS resonator’s width can go up to 6 µm with a metal-3 layer and can go up to 5.1 µm [14] after post-processing. That is why the thickness should not exceed 4 µm. Otherwise, devices cannot resonate. In this study, we set the width as 4.5 µm (Figure 1) and, hence, only three different thickness profiles were used. The thinner the beam, the larger the FS at relatively low temperature (T < 285 K) as was demonstrated in [4]. However, this behavior changed with the increase in temperature (Figure 3). The thickness has almost no effect on the FS at the bending point according to Equation (1) and COMSOL. The total absolute FS was 146.5 kHz when the beam thickness is 1 µm, and it was around 142.7 kHz when the thickness was 3 µm according to COMSOL. In the same way, it is 168.4 kHz when the thickness is 1 µm, and 164.8 mkHz when the thickness is 3 µm according to (1). Although there was no noticeable change in the FS, the thinner beam was preferable due to requiring less temperature for bending (T_bending point_ = 312 K, Figure 3b) and, consequently, has smaller thermal stresses [18]. That is why the length study is conducted for 1 µm thick beam (Device 2) in Figure 4.

The minimum length was set as 50 µm and the maximum one was set as 110 µm for the following reasons; 50 µm beam already exceeded the temperature limit (Figure 4b, T_bending point_ = 650 K) that the CMOS layers could tolerate and 110 µm beam was at the limit of stiction risk in post fab process due to sacrificing low stiffness constant. FS increased with the increase in length at relatively low temperatures (Figure 4), and this is attributed to the fact that the longer beam has higher TCF values [4,14]; however, this is only valid before the bending point. Once the beam reaches the bending point, the shorter beam results in larger FS and consequently better sensitivity. The total absolute FS increased from 169.9 kHz to 382 kHz according to COMSOL and it is increased from 184.4 kHz to 419 kHz according to (1) when the length decreased from *L* = 110 µm to *L* = 50 µm

There is no study conducted on the effect of width on temperature sensitivity because CMOS is not a custom process and the number of layers and their thicknesses are well defined. In addition, highly sensitivity temperature sensors require material with high thermal expansion constant, such as aluminum layers, and this eliminates the possibility of changing the width.

The optimum structure is shaped according to the results obtained from Figure 3 and Figure 4 with a length of 57 µm and a thickness of 1 µm (Device 2). The total absolute FS is 343 kHz (|TCF| = 589,698 ppm/K at Vth = 3.3252 V, |TCF| = 2,178,946 ppm/K at Vth = 3.3476 V) where the maximum temperature around bending point is 530 K with a 0.14 µm bending. The optimum structure’s working temperature is limited to 530 K in this work because the maximum allowable temperature for the similar structure in CMOS process was found to be around 530 K when 5.7 V and 17.4 mW was applied on embedded polysilicon layer [18]. The final structure’s mesh was set to “extremely fine mesh” with a very high-density sweep of Vth (0.0004 V resolution) to get the maximum accuracy in the results. The good match is achieved between COMSOL and (1); total absolute FS is 343 kHz according to COMSOL, and it is 356 kHz according to (1). 

The 0.14 µm thermal bending offers the potential for a high-density thermal detector array in CMOS. The total improvement of resonator’s sensitivity with respect to temperature can be derived from the ratio of the total absolute FS with Joule-heating application (X + |Y| (Figure 2b)) over the FS without any Joule-heating application (at Vth = 0 V). FS at Vth = 0 V is 11.2 kHz, and total absolute FS is 343 kHz (Figure 4b) for 57 µm long beam, and this brings around a 31× improvement in the overall sensitivity.

## 4. Conclusions

Fixed–Fixed beam type CMOS–MEMS resonator was studied in detail and optimized to build the state-of-the-art temperature sensors for high-performance uncooled microbolometers. The best performance was achieved with 57 µm long and 1 µm thick fixed–fixed beam with a maximum temperature of around 530 K, that is close but still under the critical temperature in CMOS technology [18]. The total frequency shift increased from 11 kHz to 343 kHz (31×) for 57 µm beam with much larger |TCF| (2,178,946 ppm/K) while keeping the pull-in force application as small as possible. Furthermore, the nonlinearity problem of fixed–fixed beam type CMOS–MEMS resonator was addressed by operating the device around the beam bending point. A good match between COMSOL and theory was demonstrated and can be used as guidance in future researches to build an ultra-sensitive temperature sensor for microbolometers in CMOS technology. This in return, can enable a less expensive, compact, and wider range of application compatibility such as internet of things.

## Figures and Tables

**Table 1:** Performance comparison between this work and literature. TCF–temperature coefficient of frequency, CMOS–complementary metal-oxide semiconductor, MEMS–Micro-Electro-Mechanical Systems, NEMS–Nano Electromechanical Systems.

Design	Resonance Frequency	Absolute |TCF| (ppm/K)	Technology
This work (57 µm long CMOS–MEMS Resonator)	1.92 MHz	2,178,946	CMOS–MEMS
120 µm long CMOS–MEMS Resonator [4]	640 kHz	4537	CMOS–MEMS
AIN Piezoelectric Nanomechanical Resonator [5]	161.4 MHz	30	NEMS
Nanomechanical Torsional Resonator [6]	842 kHz	548	NEMS
Silicon Micromechanical Resonator [7]	101 MHz	29.7	MEMS
